# Interface modification effect between p-type a-SiC:H and ZnO:Al in p-i-n amorphous silicon solar cells

**DOI:** 10.1186/1556-276X-7-81

**Published:** 2012-01-18

**Authors:** Seungsin Baek, Jeong Chul Lee, Youn-Jung Lee, Sk Md Iftiquar, Youngkuk Kim, Jinjoo Park, Junsin Yi

**Affiliations:** 1 School of Information and Communication Engineering, Sungkyunkwan University, 300 Cheoncheon-dong, Jangan-gu, Suwon, 440-746, South Korea; 2 Photovoltaic Research Center, Korea Institute of Energy Research, 152 Gajeong-ro, Yuseong-gu, Daejeon, 305-343, South Korea

**Keywords:** buffer layer, amorphous materials, thin films, plasma deposition, electrical properties

## Abstract

Aluminum-doped zinc oxide (ZnO:Al) [AZO] is a good candidate to be used as a transparent conducting oxide [TCO]. For solar cells having a hydrogenated amorphous silicon carbide [a-SiC:H] or hydrogenated amorphous silicon [a-Si:H] window layer, the use of the AZO as TCO results in a deterioration of fill factor [FF], so fluorine-doped tin oxide (Sn0_2_:F) [FTO] is usually preferred as a TCO. In this study, interface engineering is carried out at the AZO and p-type a-SiC:H interface to obtain a better solar cell performance without loss in the FF. The abrupt potential barrier at the interface of AZO and p-type a-SiC:H is made gradual by inserting a buffer layer. A few-nanometer-thick nanocrystalline silicon buffer layer between the AZO and a-SiC:H enhances the FF from 67% to 73% and the efficiency from 7.30% to 8.18%. Further improvements in the solar cell performance are expected through optimization of cell structures and doping levels.

## Introduction

TCO plays an important role in a silicon-based thin-film solar cell because of good electrical conductivity as well as optical properties such as transparency and haze ratio. Additionally, the interface properties with an adjacent p-layer are also important. The FTO has been widely used as a front TCO in a single p-i-n a-Si:H solar cell. The FTO, however, has a low haze ratio in a wavelength longer than 700 nm, which is a limiting factor for tandem solar cells that incorporates a low-bandgap bottom cell. The AZO has several advantages such as good electrical conductivity, plasma robustness, light-scattering properties [1-3], etc. One drawback of AZO is that when it is used in amorphous silicon [a-Si] solar cells, having a-SiC:H or a-Si:H window layer, the FF deteriorates [4-7]. It is assumed that the work function of AZO is lower than that of FTO [8], causing an increased barrier potential to occur at the front interface obstructing the carrier movements and thus, lowering the FF. In this paper, we report some attempts in interface engineering to lower the effect of the barrier between the AZO and the p-type a-SiC:H in order to enhance the FF and efficiency.

## Experimental methods

**Figure 1:**
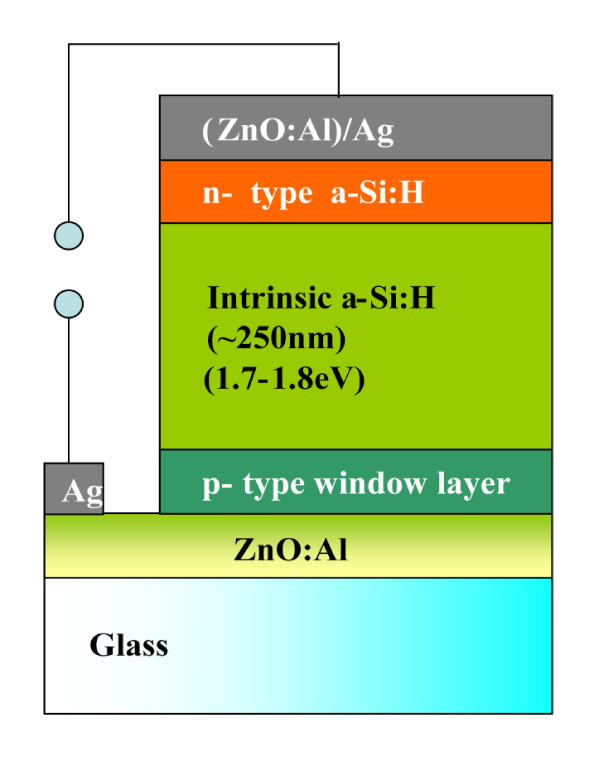
**The p-i-n a-Si:H solar cell structure using AZO as TCO**.

**Figure 2:**
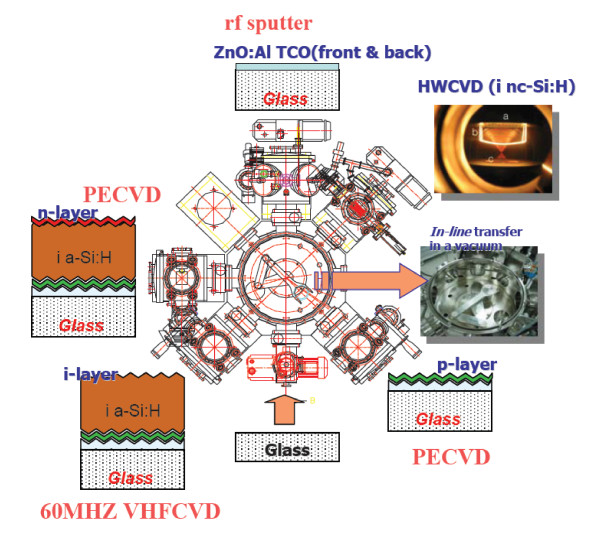
**The cluster-type multi-chamber system**.

The structure of the p-i-n a-Si solar cells is shown in Figure 1. The textured AZO films are prepared by chemical etching of radiofrequency [rf] magnetron-sputtered AZO, in 1% HCl solution. The base pressure for the rf sputtering is 1 × 10^-7 ^Torr. The AZO deposition pressure is set at 5 mTorr with Ar flow rate as 3 sccm. An rf power of 100 W is used in the AZO deposition. For p-i-n silicon film deposition, a cluster-type multi-chamber chemical vapor deposition [CVD] system is used, as shown in Figure 2. Each of the solar cell layers is prepared in a separate chamber. For the deposition of p- and n-type silicon films, a conventional 13.56-MHz capacitively coupled CVD is used with SiH_4_, H_2_, B_2_H_6 _(1% in H_2_), CH_4 _(50% in H_2_), and PH_3 _(1% in H_2_) gases. The intrinsic a-Si:H layer is deposited with a 60-MHz very-high-frequency CVD with SiH_4 _and H_2 _gases. The dilution ratios, *R *= H_2_/SiH_4_, are 8 and 0.8 for i-type a-Si:H and p-type a-SiC:H, respectively. The substrate temperature and deposition pressure are kept at 200°C and 0.3 Torr, respectively, for all silicon layers. Dark *I*-*V *characteristics are measured, and the applied bias-dependent TCO/p series resistance is estimated.

Interface modification of AZO/p-type a-SiC:H is carried out by inserting a highly conductive material between the AZO and p-type a-SiC:H as a buffer layer. It is first done by profiling the flow rate of B_2_H_6 _during the deposition of p-type a-SiC:H. Initially, the flow rate of B_2_H_6 _is kept high to minimize the depletion in the p-layer, and then, the flow rate of B_2_H_6 _is decreased to reduce the light absorption. After the B_2_H_6 _profiling, the cell results in a AZO/p+ a-SiC:H/p a-SiC:H/i a-Si:H/n a-Si:H structure. In another set, a nanocrystalline silicon layer is used as the buffer layer. A few-nanometer-thick nc-Si:H is deposited on AZO before the cell fabrication. All investigated cells have a 500-nm Ag back contact that defines a 0.25-cm^2 ^cell area. The illuminated *I*-*V *properties of solar cells are measured by an AM 1.5 G double-beam solar simulator (Wacom, Co., Ltd, Kazo-shi, Saitama, Japan), and series resistance is obtained from the *I*-*V *curves.

## Results and discussion

**Figure 3:**
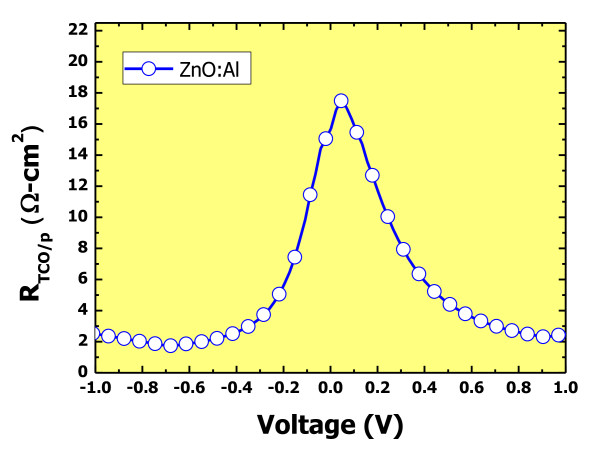
**The dependence of resistance of AZO/p-type a-SiC:H interface on the applied voltage**.

Figure 3 shows the voltage-dependent series resistance for AZO/p-type a-SiC:H structures. It is seen that the resistance is highly dependent on the applied voltage. The resistance displays a Gaussian distribution centered at 0 V, and the peak value is as high as 18 Ω cm^2^. It implies that there exists some kind of obstruction of carrier movement which is dependent on the applied voltage. It seems that the obstruction is most likely the interface barrier caused by the work function differences between the AZO and p-type a-SiC:H.

**Figure 4:**
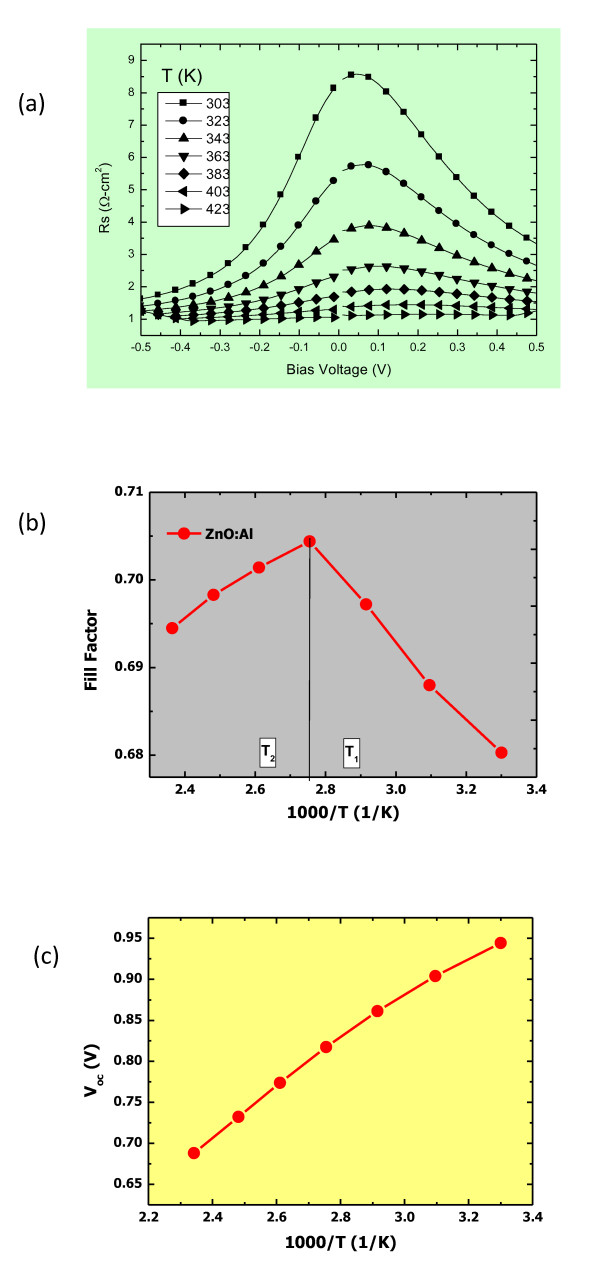
**Variation of resistance (*R _TCO/p_*), FFs and *V*_oc_**. (**a**) The bias voltage dependence of ***R_TCO/p_***under various temperatures. (**b**) The temperature dependence of FFs for solar cell. (**c**) The temperature dependence of *V*_oc _for cells with the AZO as a TCO.

Figure 4a shows the dependence of resistance on the bias voltage as the temperature is increased. As the temperature increases, the resistance decreases and so does its dependence on the bias voltage. At 0 V, the *R*_TCO/p _remains below 2.5 Ω cm^2 ^when the temperature is over 363 K. Figure 4b, c shows the dependence of solar cell characteristics on the measured temperature of p-i-n a-Si solar cells with AZO as TCO. Here, the temperature range has been divided into two regions: T_1 _and T_2_, as shown in Figure 4b. In region T_2 _(above 343 K), the FF decreases as the temperature increases, which is in accordance with the generally known fact that the FF decreases with an increased temperature due to the leakage current. However, in region T_1 _(303 to 343 K), a different behavior of FF is observed. In this range of temperature, the FF decreases rapidly with a decrease in temperature. As the temperature is lowered, the junction potential at the interface increases, which may be the dominant factor for the decrease in the FF. Figure 4c shows that the open-circuit voltage [*V*_oc_] increases inversely with the temperature *T*, which we find that *V*_oc _decreases linearly with the temperature. Thus, it becomes necessary and possible to improve the FF in a-Si solar cell where AZO is used as a TCO.

**Figure 5:**
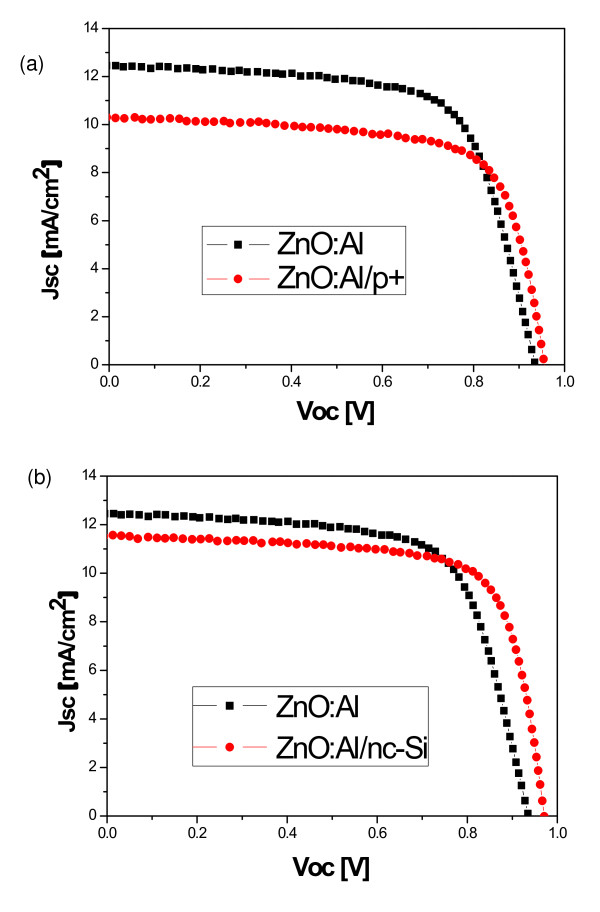
**Light *I*-*V *characteristics**. The light *I*-*V *characteristics of solar cells having (**a**) AZO/p-type a-SiC:H and AZO/p+ a-SiC:H/p-type a-SiC:H front interface and (**b**) AZO/p-type a-SiC:H and AZO/nc-Si/p-type a-SiC:H front interface.

As the potential barrier at the AZO/p interface is due to the work function difference, it is possible to insert a suitable buffer layer, with higher conductivity, between the AZO and p-type a-SiC:H in order to reduce the loss in the FF. As mentioned in the 'Experimental methods' section, a p+ a-SiC:H buffer layer is made between the AZO and p-type a-SiC:H by profiling the B_2_H_6 _flow rate. Figure 5a shows light *I*-*V *characteristics of the cells. With the introduction of the buffer layer, there is an increase in the *V*_oc_, but a reduction in the short-circuit current density [*J*_sc_] has also been observed. The increased conductivity of the p+ a-SiC:H buffer layer reduces the effect of the potential barrier, but the increased doping concentration results in increased defect density in the p+ a-SiC:H and the interfaces, thus, more carrier recombination and decreased *J*_sc_. Figure 5b shows the light *I*-*V *characteristics of AZO/p-type a-SiC:H and AZO/buffer/p-type a-SiC:H solar cells where the buffer layer is a few-nanometer-thick nanocrystalline silicon. Even though there is a smaller decrease in the *J*_sc_, it can be seen that the *V*_oc _and FF are much more improved

**Figure 6:**
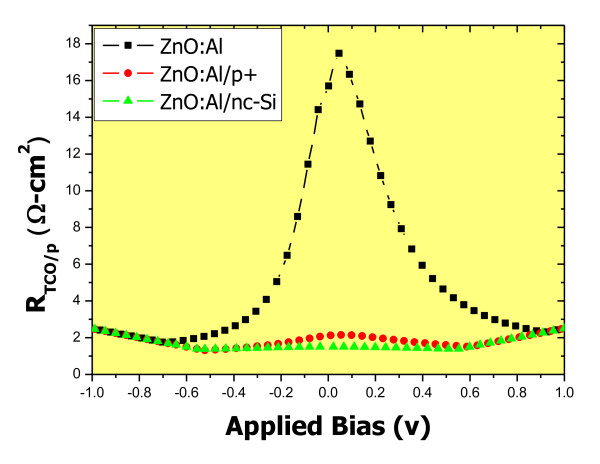
**Dependence of resistance on the applied bias**. The dependence of resistance on the applied bias for a normal cell with AZO as TCO and two other cells with interface engineering.

It has been shown that the resistance of the AZO/p-type a-SiC:H p-i-n solar cell is highly dependent on the bias voltage. The maximum resistance is obtained with the bias of around 0 V. It is assumed that there is a potential barrier at the interface which disturbs the carrier movement, and the height of the potential barrier is affected by applied voltage. To reduce the effect of the barrier at the interface, buffer layers are inserted. Figure 6 shows the bias-dependent resistance of the AZO/p-type a-SiC:H p-i-n solar cell and the ones with interface engineering. It is seen that the resistance for the cells with the buffer layers does not depend much on the applied voltage, indicating that the effect of potential barrier at the interface is almost negligible. Although the cell with the AZO/p+ a-SiC:H/p-type a-SiC:H front interface structure does not show the effect of potential barrier at the interface, its solar cell characteristics are not improved. It means that just by getting rid of the potential barrier does not improve the efficiency of solar cells. Good electrical, optical, and interface properties as well as lowering the interface potential barrier are necessary to assure high cell efficiency.

**Figure 7:**
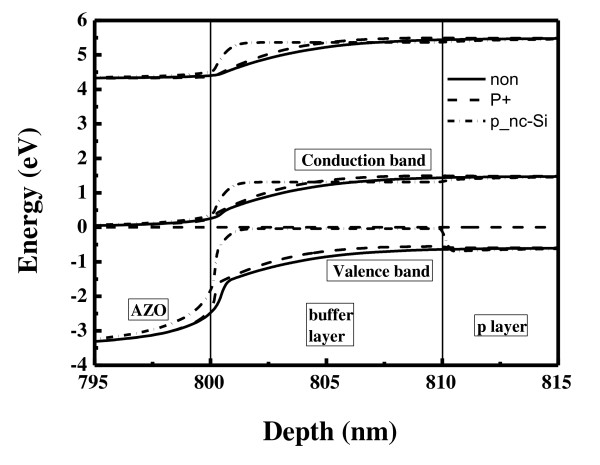
**Energy band diagram**. Energy band diagram of the interface between the AZO and p-type layer with and without the buffer layers.

Figure 7 shows the band diagrams of the interface between the AZO and p-layer with and without the buffer layers simulated by ASA. At the depth of about 801 nm, it is seen that although the barrier height at the valence band actually decreases for the one with a p+ buffer layer, the shape of the energy band diagram is similar to the one without a buffer layer. For the case where nanocrystalline silicon is used as a buffer layer, the shape of the energy band diagram changes much. First, the barrier height is reduced. The bandgap of the nanocrystalline silicon is smaller than a-SiC:H, indicating that both the optical and electrical properties are different. At the depth of about 810 nm, because of the smaller energy bandgap of the nanocrystalline silicon, an opposite banding occurs at the valence band. It helps generated holes to move towards the TCO and be collected.

Table 1 shows that the FF and *V*_oc _improve when the potential barrier is lowered; this is a different set of cells than that in Figure 5. There is a large drop in *J*_sc _in the case where p+ a-SiC:H is used as a buffer layer due to increased defects and lower optical bandgap of this layer. When the nanocrystalline silicon layer is used as a buffer layer, there is a large increase in the *V*_oc _and FF with a smaller decrease in the *J*_sc_, resulting in higher cell efficiency.

**Table 1:** Solar cell parameters for cells with and without the interface engineering

	AZO	AZO/p+	AZO/nc-Si
*V*_oc _(V)	0.92	0.94	0.97
*J*_sc _(mA/cm^2^)	11.7	10.3	11.53
FF	0.67	0.71	0.73
Efficiency (%)	7.30	7.01	8.18

*V*_oc_, open-circuit voltage; *J*_sc_, short-circuit current density; FF, fill factor; AZO, ZnO:Al.

There are a number of factors that limit the solar cell performance, the potential barrier at the front interface being one of them. Since the material properties of nanocrystalline silicon differ from the amorphous silicon, its electrical characteristics such as electron affinity, mobility gap, electrical conductivities, etc. also differ, and some are favorable for the solar cells. That is why the solar cell performance with the nc-Si as a buffer layer is much better. In this paper, improvements of a-Si p-i-n solar cell with AZO as TCO have been obtained only by engineering the interface between the p-type a-SiC:H and AZO and shows that a large increase in the solar cell efficiency can be obtained by using nc-Si as a buffer layer. In this study, no other optimizations such as optimizing the thickness of each layer, etc. have been carried out.

## Conclusion

In order to improve the FF of a-Si p-i-n solar cells with AZO as TCO, interface engineering has been carried out by inserting a buffer layer between the AZO and p-type a-SiC:H. It is shown that by engineering the interface between the two materials, the effect of potential barrier at the interface can be reduced, resulting in an increase in the FF and cell efficiency. The best cell performance obtained in this study is by using nc-Si as a buffer layer since it provides better optical transmission and electrical conductivity suitable for the solar cell performance. The cell efficiency improves from 7.30% to 8.18% by using a few-nanometer-thick nc-Si layer. Further improvements in the solar cell performance are possible through optimization of thickness of each layer, doping concentration, etc.

## Competing interests

The authors declare that they have no competing interests.

## Authors' contributions

JCL gave an idea for related thin-film solar cell analysis and worked on the manuscript. Y-JL also contributed to the experiment analysis with scientific opinion. SMI had focused on the related Si thin-film solar cell devices with SB. YK also generated the general analysis for the thin-film solar cell as well as the single-layer of amorphous. JP mainly focused on the analysis of the basis on the solar cell as well as the overall collection and arranged the optimized manuscript. JY had been guiding the research. All authors read and approved the final manuscript.
